# Endothelial dysfunction is an early indicator of sepsis and neutrophil degranulation of septic shock in surgical patients

**DOI:** 10.1002/bjs5.50265

**Published:** 2020-02-19

**Authors:** M. Martin‐Fernandez, L. M. Vaquero‐Roncero, R. Almansa, E. Gómez‐Sánchez, S. Martín, E. Tamayo, M. C. Esteban‐Velasco, P. Ruiz‐Granado, M. Aragón, D. Calvo, J. Rico‐Feijoo, A. Ortega, E. Gómez‐Pesquera, M. Lorenzo‐López, J. López, C. Doncel, C. González‐Sanchez, D. Álvarez, E. Zarca, A. Ríos‐Llorente, A. Diaz‐Alvarez, E. Sanchez‐Barrado, D. Andaluz‐Ojeda, J. M. Calvo‐Vecino, L. Muñoz‐Bellvís, J. I. Gomez‐Herreras, C. Abad‐Molina, J. F. Bermejo‐Martin, C. Aldecoa, M. Heredia‐Rodríguez

**Affiliations:** ^1^ Group for Biomedical Research in Sepsis (BioSepsis) Instituto de Investigación Biomédica de Salamanca (IBSAL) Salamanca Spain; ^2^ Anaesthesiology and Reanimation Service Hospital Universitario de Salamanca Salamanca Spain; ^3^ Department of General and Gastrointestinal Surgery Hospital Universitario de Salamanca, Instituto de Investigación Biomédica de Salamanca (IBSAL) and Universidad de Salamanca Salamanca Spain; ^4^ Research Unit Hospital Clínico Universitario de Valladolid Valladolid Spain; ^5^ Group for Biomedical Research in Critical Care (BioCritic), Anaesthesiology and Reanimation Service Hospital Clínico Universitario de Valladolid Valladolid Spain; ^6^ Clinical Analysis Service Hospital Clínico Universitario de Valladolid Valladolid Spain; ^7^ Intensive Care Medicine Service Hospital Clínico Universitario de Valladolid Valladolid Spain; ^8^ Microbiology and Immunology Service Hospital Clínico Universitario de Valladolid Valladolid Spain; ^9^ Anaesthesiology and Reanimation Service Hospital Universitario Río Hortega Valladolid Spain; ^10^ Biomedical Research Networking Centre on Respiratory Diseases (CIBERES), Instituto de Salud Carlos III Madrid Spain; ^11^ Biomedical Research Networking Centre on Cancer (CIBERONC) Madrid Spain

## Abstract

**Background:**

Stratification of the severity of infection is currently based on the Sequential Organ Failure Assessment (SOFA) score, which is difficult to calculate outside the ICU. Biomarkers could help to stratify the severity of infection in surgical patients.

**Methods:**

Levels of ten biomarkers indicating endothelial dysfunction, 22 indicating emergency granulopoiesis, and six denoting neutrophil degranulation were compared in three groups of patients in the first 12 h after diagnosis at three Spanish hospitals.

**Results:**

There were 100 patients with infection, 95 with sepsis and 57 with septic shock. Seven biomarkers indicating endothelial dysfunction (mid‐regional proadrenomedullin (MR‐ProADM), syndecan 1, thrombomodulin, angiopoietin 2, endothelial cell‐specific molecule 1, vascular cell adhesion molecule 1 and E‐selectin) had stronger associations with sepsis than infection alone. MR‐ProADM had the highest odds ratio (OR) in multivariable analysis (OR 11·53, 95 per cent c.i. 4·15 to 32·08; *P* = 0·006) and the best area under the curve (AUC) for detecting sepsis (0·86, 95 per cent c.i. 0·80 to 0·91; *P* < 0·001). In a comparison of sepsis with septic shock, two biomarkers of neutrophil degranulation, proteinase 3 (OR 8·09, 1·34 to 48·91; *P* = 0·028) and lipocalin 2 (OR 6·62, 2·47 to 17·77; *P* = 0·002), had the strongest association with septic shock, but lipocalin 2 exhibited the highest AUC (0·81, 0·73 to 0·90; *P* < 0·001).

**Conclusion:**

MR‐ProADM and lipocalin 2 could be alternatives to the SOFA score in the detection of sepsis and septic shock respectively in surgical patients with infection.

## Graphical abstract

In surgical patients, endothelial dysfunction is an early indicator differentiating sepsis from simple infection, whereas neutrophil degranulation is an early indicator differentiating septic shock from sepsis. Mid‐regional proadrenomedullin and lipocalin 2 are the most representative biomarkers of sepsis and septic shock respectively.

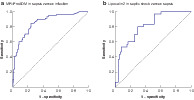

Useful biomarkers that merit validation elsewhere.

## Abstract (es)

**Antecedentes:**

La estratificación de la gravedad de una infección se basa actualmente en la puntuación SOFA (*Sequential Organ Failure Assessment*), que es difícil de calcular fuera de la unidad de cuidados intensivos. Los biomarcadores podrían ayudar a estratificar la gravedad de la infección en pacientes quirúrgicos.

**Métodos:**

Se compararon las concentraciones de 10 biomarcadores que denotan disfunción endotelial, 22 que indican granulopoyesis de emergencia y 6 que expresan la degranulación de neutrófilos en tres grupos de pacientes de tres hospitales españoles (100 con infección, 95 con sepsis y 57 con shock séptico) en las primeras doce horas después del diagnóstico.

**Resultados:**

Siete biomarcadores que expresan disfunción endotelial (proadrenomedulina, sindecan‐1, trombomodulina, angiopoyetina‐2, endocan‐1, molécula de adhesión endotelial 1 y E‐selectina) mostraron una fuerte asociación con la sepsis en comparación con la infección aislada. La proadrenomedulina presentó el valor más alto de la razón de oportunidades (*odds ratio*, OR) en el análisis multivariable (OR 11,53, i.c. del 95% 4,15‐32,08, *P* = 0,006) y la mejor área bajo la curva para detectar sepsis (AUC 0,86, i.c. del 95% 0,80‐0,91, *P* < 0,001). En la comparación entre sepsis y shock séptico, los biomarcadores que mostraron la asociación más estrecha con el shock séptico fueron dos biomarcadores de degranulación de neutrófilos (proteinasa‐3 y lipocalina‐2) (OR 8,09, i.c. del 9% 1,34‐48,91, *P* = 0,028; OR 6.62, i.c. del 95% 2,47‐17,77, *P* = 0,002), pero la lipocalina‐2 presentó la mejor AUC (0,81, i.c. del 95% 0,73‐0,90, *P* < 0,001).

**Conclusión:**

la proadrenomedulina y la lipocalina‐2 podrían representar alternativas a la puntuación SOFA para detectar sepsis y shock séptico en pacientes quirúrgicos con infección.

## Introduction

Sepsis and septic shock are major causes of morbidity and mortality in surgical patients^1^. In a patient with infection, prompt detection of sepsis is key to the initiation of early treatment with appropriate antimicrobials, elimination of the infectious source, administration of fluids and appropriate transfer to the ICU. In patients with sepsis, prompt detection of septic shock could imply a need to modify antibiotic treatment, seek alternative sources of

potentially infectious organisms not already identified, and adjust ICU support. Since publication of the Third International Consensus Definitions for Sepsis and Septic Shock (SEPSIS‐3) in 2016^2^, severity stratification in patients with infection has been based on the Sequential Organ Failure Assessment (SOFA) score^3^. The problem with this score is that it is difficult to calculate in non‐ICU settings, such as surgical departments or the emergency room. The alternative proposed by the SEPSIS‐3 consensus for these settings, the quickSOFA (composed of three simple items: respiratory frequency, BP and the Glasgow Coma Scale score), is very specific but less useful for detecting sepsis^4^.

Biomarkers could contribute to stratification of the severity of infection. Sepsis is characterized by acute endothelial dysfunction, which increases vascular permeability, promotes activation of the coagulation cascade and tissue oedema, and compromises the perfusion of vital organs^5^. Biomarkers of endothelial responses can be used to categorize patients into homogeneous subgroups with different severity^6^. In turn, sepsis activates emergency granulopoiesis, inducing release of immature neutrophil precursor cells in the peripheral blood, an event related directly to severity^7^, ^8^, ^9^, ^10^. Emergency granulopoiesis can be detected by profiling the mRNA in blood of the genes that are expressed sequentially in the neutrophil precursors^11^, ^12^. Other molecules denoting severity during an infection are proteins released to the plasma during neutrophil degranulation^13^, ^14^. These include matrix metalloproteinase (MMP) 8, neutrophil gelatinase‐associated lipocalin and lactotransferrin, which have been shown to be closely related to the development of sepsis^15^, and levels of plasma MMPs 3, 7, 8 and 9 are increased in severe sepsis on admission to the ICU^16^.

In this study, 38 biomarkers of endothelial dysfunction, emergency granulopoiesis or neutrophil degranulation were evaluated to stratify severity in surgical patients with infection. The hypothesis was that these biomarkers might differentiate between three groups of patients: those with infection, those with sepsis, and those with septic shock.

## Methods

Surgical patients with infection, sepsis or septic shock were recruited prospectively from the surgery departments and surgical ICUs of the three participating hospitals (Hospital Clínico Universitario de Valladolid, Hospital Universitario Río Hortega de Valladolid and Hospital Clínico Universitario de Salamanca), between January 2017 and January 2019. Infection was defined according to the US Centers for Disease Control and Prevention National Surveillance Definitions for Specific Types of Infections^17^. Sepsis and septic shock were defined using the SEPSIS‐3 consensus definitions^2^, ^18^. A specific standard survey was employed in the three participating hospitals to collect clinical data along with results of haematological, biochemical, radiological and microbiological investigations. Healthy controls with similar age and sex characteristics to the patients were recruited from the Centro de Hemoterapia y Hemodonación de Castilla y León (CHEMCYL, Valladolid, Spain).

### Ethical approval

The study was approved by the respective Committees for Ethics in Clinical Research of the three participating hospitals. Methods were carried out in accordance with current Spanish law for Biomedical Research, fulfilling the standards indicated by the Declaration of Helsinki. Written informed consent was obtained from patients' relatives or their legal representative before enrolment.

### Microbiology

Standard cultures in biological samples, guided by the presumptive source of the infection, were performed to assess the presence of the causal pathogen. Potentially contaminant microorganisms were not considered.

### Biomarker profiling

Thirty‐eight biomarkers (10 denoting endothelial dysfunction, 22 indicating emergency granulopoiesis and 6 denoting neutrophil degranulation) were profiled in the three patient groups (infection, sepsis or septic shock) in the first 12 h after diagnosis (*Tables* *S1* and *S2*, supporting information). The methods used to profile these biomarkers are detailed in *Appendix* *S1* (supporting information). Blood from healthy individuals was collected as part of their blood donation.

### Statistical analysis

Statistical analysis was performed with IBM SPSS**®** version 20 (IBM, Armonk, New York, USA). Box plots were represented using Minitab**®** 19.2 (Minitab, Coventry, UK). For demographic and clinical characteristics of the patients, differences between groups were assessed using the χ^2^ test for categorical variables. Differences between groups for continuous variables were assessed with the Kruskal–Wallis test, with *post hoc* tests adjusting for multiple comparisons. In the comparison of infection and sepsis, multivariable logistic regression analysis was employed to evaluate the association between biomarker levels and the presence of sepsis. In the comparison of sepsis and septic shock, the same type of analysis was employed to evaluate the association between biomarker levels and the presence of septic shock. Only biomarkers yielding *P* ≤ 0·050 in univariable analysis were tested in multivariable analyses. Potential confounding clinical factors that yielded *P* ≤ 0·100 in univariable analysis were introduced as adjusting variables in multivariable analyses, followed by multiple testing correction by the false discovery rate using the Benjamini–Hochberg procedure. The optimal operating point in the area under the curve (AUC) analysis was identified as described previously^19^.

## Results

There was a total of 100 patients with infection, 95 with sepsis and 57 with septic shock. Patients with infection were significantly younger than those in the other groups (*Table* 1), and the healthy controls. Proportions of men to women were similar in all patient groups and control subjects. Patients with sepsis and septic shock had more antecedent cardiovascular, respiratory or renal disease. The proportion of patients needing urgent surgery was similar in the three groups. Abdominal surgery was the most frequent type, and the abdomen was the predominant source of infection in all three patient groups.

**Table 1:** Clinical characteristics of the patients

	Infection (*n* = 100)	Sepsis (*n* = 95)	Septic shock (*n* = 57)	*P* † (infection *versus* sepsis)	*P* † (infection *versus* septic shock)	*P* † (sepsis *versus* septic shock)
**Age (years)** *	57·0 (39·25–70·50)	73·0 (59–80)	74·0 (68–78·5)	< 0·001‡	< 0·001‡	1·000
**Male sex**	60 (60·0)	62 (65)	31 (54)	0·448	0·493	0·183
**Co‐morbidity**						
Chronic cardiovascular disease	13 (13·0)	28 (29)	21 (37)	0·005	< 0·001	0·347
Chronic respiratory disease	2 (2)	13 (14)	6 (11)	0·003	0·022	0·569
High BP	30 (30·0)	47 (49)	34 (60)	0·009	< 0·001	0·223
Chronic renal failure	2 (2·0)	9 (9)	7 (12)	0·024	0·004	0·388
Chronic hepatic failure	4 (4·0)	2 (2)	1 (2)	0·422	0·423	0·880
Diabetes mellitus	14 (14·0)	20 (21)	17 (30)	0·194	0·017	0·222
Cancer	13 (13·0)	18 (19)	13 (23)	0·256	0·112	0·568
Immunosuppression	5 (5·0)	13 (14)	6 (11)	0·037	0·205	0·553
**Surgery type**						
Urgent	70 (70·0)	74 (78)	41 (72)	0·210	0·798	0·407
Abdominal	65 (65·0)	54 (57)	24 (42)	0·243	0·005	0·078
Cardiothoracic	0 (0)	14 (15)	14 (25)	< 0·001	< 0·001	0·130
Vascular	1 (1·0)	3 (3)	1 (2)	0·288	0·685	0·601
Urological/renal	0 (0)	0 (0)	1 (2)	§	0·184	0·195
Other	13 (13·0)	4 (4)	4 (7)	0·030	0·246	0·453
**Time course and outcome**						
Length of hospital stay (days)*	5 (2–12)	15 (8–29·5)	31 (18·50–48·75)	< 0·001‡	< 0·001‡	< 0·001‡
Length of ICU stay (days)*	0·5 (0–2)	3 (1–6·75)	7 (3–13)	< 0·001‡	< 0·001‡	0·001‡
Hospital mortality	0 (0)	7 (7)	22 (39)	0·006	< 0·001	< 0·001
**Source of infection**						
Respiratory tract	4 (4·0)	15 (16)	14 (25)	0·006	< 0·001	0·183
Abdomen	67 (67·0)	48 (51)	19 (33)	0·019	< 0·001	0·039
Urinary tract	0 (0)	4 (4)	6 (11)	0·038	0·001	0·128
Surgical site	12 (12·0)	16 (17)	13 (23)	0·335	0·075	0·365
Bacteraemia	0 (0)	6·30 (6)	12 (21)	0·011	< 0·001	0·006
Other	13 (13·0)	16 (17)	4 (7)	0·451	0·246	0·083
**Microbiology**						
Positive culture	29 (29·0)	54 (57)	43 (75)	< 0·001	< 0·001	0·021
Gram‐positive	13 (13·0)	29 (31)	21 (37)	0·003	< 0·001	0·422
Gram‐negative	23 (23·0)	31 (33)	34 (60)	0·133	< 0·001	0·001
Fungal	4 (4·0)	8 (8)	8 (14)	0·199	0·023	0·275
**Measurements at diagnosis** *						
SOFA score	0 (0–1)	6 (3–8)	9 (7–11)	< 0·001‡	< 0·001‡	< 0·001‡
Total bilirubin (mg/dl)	0·70 (0·4–1·03)	0·70 (0·43–1·78)	1·00 (0·56–1·89)	1·000‡	0·013‡	0·072‡
Glucose level (mg/dl)	118 (105–140)	158 (117·5–184)	163 (128–232·50)	< 0·001‡	< 0·001‡	1·000‡
Sodium level (mmol/l)	139·00 (136–141·25)	138·00 (135–141·25)	129·00 (134–142)	0·008‡	< 0·001‡	0·001‡
Potassium level (mmol/l)	3·90 (3–4·10)	4·00 (3·50–4·20)	3·85 (3–4·12)	§	§	§
Platelet count (cells/mm^3^)	221 500 (186 250–299 250)	184 000 (105 250–276 000)	123 000 (88 500–258 000)	0·023‡	0·001‡	0·794‡
INR	1·15 (1·03–1·26)	1·27 (1·16–1·35)	1·44 (1·27–1·81)	0·002‡	< 0·001‡	< 0·001‡
Albumin (mg/dl)	3480 (2737·5–4135)	2445 (2132·50–3130)	2340 (1837·5–2752·5)	< 0·001‡	< 0·001‡	1·000‡
Lactate (mmol/l)	1·50 (1·06–1·85)	1·50 (1·23–2)	3·55 (2·46–5·19)	1·000‡	< 0·001‡	< 0·001‡
White blood cell count (cells/mm^3^)	13 070 (9187·5–16 347·5)	14 050 (9410–18 290)	14 550 (7525–19 880)	§	§	§
Lymphocytes (cells/mm^3^)	1383·50 (911·50–1806·36)	940 (600·10–1453·86)	592 (410·08–1103·39)	0·021‡	< 0·001‡	0·004‡
Monocytes (cells/mm^3^)	795·52 (466·07–1099)	632 (345–962)	411·25 (245–791·56)	0·049‡	0·002‡	0·196‡
Neutrophils (cells/mm^3^)	10 144 (6327·70–13 864·48)	12 100 (7857–15 195)	12 240 (5647·50–18 177·50)	0·091‡	0·199‡	1·000‡
Eosinophils (cells/mm^3^)	43 (12·50–117·41)	19 (0–57·52)	10·92 (0–47·53)	§	§	§
Basophils (cells/mm^3^)	33·60 (18–59·73)	26·90 (11·76–54·80)	20 (10·54–49·92)	0·827‡	0·042‡	0·196‡

Values in parentheses are percentages unless indicated otherwise;

* values are median (i.q.r.). SOFA, Sequential Organ Failure Score; INR, international normalized ratio.

† χ^2^ test, except

‡ Kruskal–Wallis test;

§ absence of *P* value for χ^2^ or Kruskal–Wallis test, as appropriate.

Respiratory infection was more common in patients with sepsis or septic shock than in patients with infection alone. The prevalence of bacteraemia was highest in patients with septic shock, where Gram‐negative bacteria dominated (*Table* 1).

Patients with septic shock showed the highest degree of organ failure as assessed by the SOFA score. Duration of hospital stay was directly associated with severity. No patient in the infection group died in hospital, compared with seven of 95 (7 per cent) patients with sepsis and 22 of 57 (39 per cent) with septic shock (*Table* 1).

**Figure 1:**
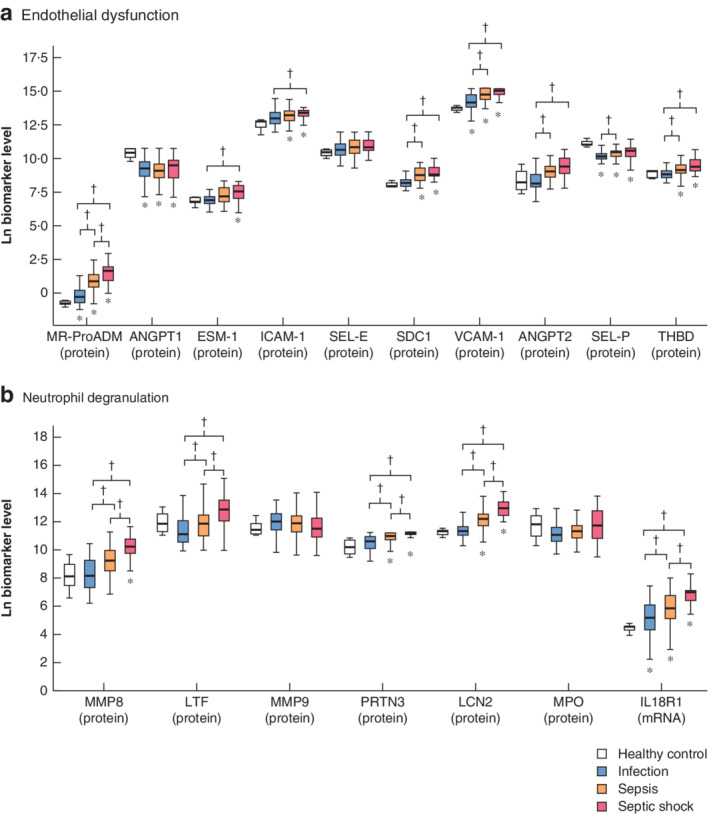
Levels of endothelial dysfunction and neutrophil degranulation biomarkers in healthy control, infection, sepsis and septic shock groups Biomarkers of **a** endothelial dysfunction and **b** neutrophil degranulation. Levels of mid‐regional proadrenomedullin (MR‐ProADM) are in nmol/l, those of interleukin‐18 receptor type 1 (IL18R1) are copies of cDNA per ng mRNA, and those of the remaining biomarkers are in pg/ml. ANGPT, angiopoietin; ESM, endothelial cell‐specific molecule; ICAM, intercellular adhesion molecule; SEL‐E, E‐selectin; SDC, syndecan; VCAM, vascular cell adhesion molecule; SEL‐P, P‐selectin; THBD, thrombomodulin; MMP, matrix metalloproteinase; LTF, lactoferrin; PRTN, proteinase; LCN, lipocalin; MPO, myeloperoxidase. *P ≤ 0·050 *versus* healthy control; †*P* ≤ 0·050 (Kruskal–Wallis test).

**Figure 2:**
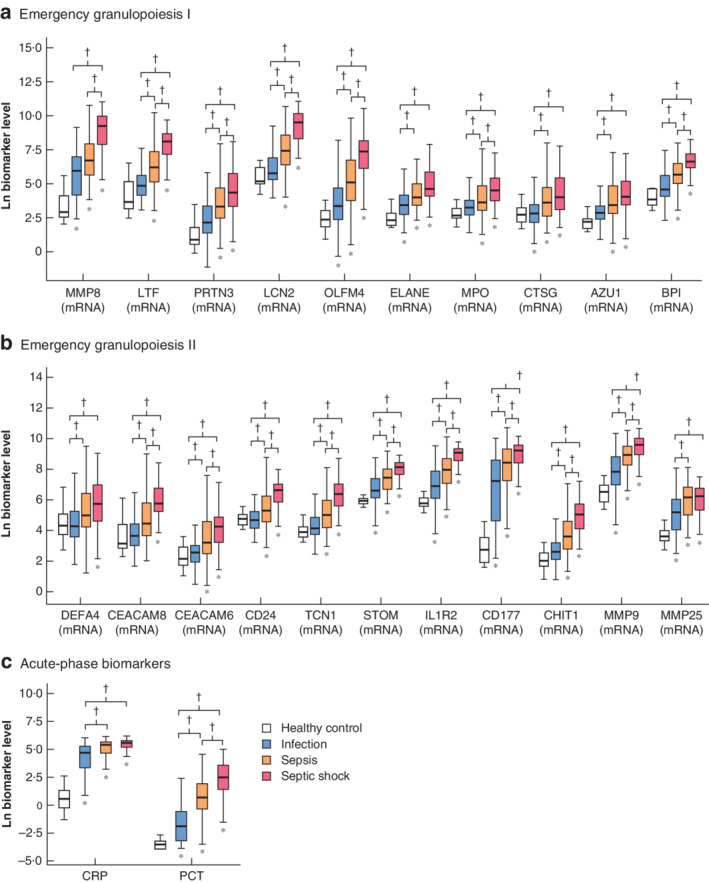
Levels of emergency granulopoiesis and acute‐phase response biomarkers in healthy control, infection, sepsis and septic shock groups Biomarkers of **a,b** emergency granulopoiesis and **c** acute‐phase response. Levels of C‐reactive protein (CRP) are in mg/l, those of procalcitonin (PCT) are in ng/ml, and those of the remaining biomarkers are copies of cDNA per ng mRNA. MMP, matrix metalloproteinase; LTF, lactoferrin; PRTN, proteinase; LCN, lipocalin, OLFM, olfactomedin; ELANE, elastase, neutrophil expressed; MPO, myeloperoxidase; CTSG, cathepsin G; AZU, azurocidin; BPI, bactericidal/permeability‐increasing protein; DEFA, defensin α; CEACAM, carcinoembryonic antigen‐related cell adhesion molecule; CD, cluster of differentiation; TCN, transcobalamin; STOM, stomatin; IL1R2, interleukin‐1 receptor type 2; CHIT, chitinase. *P ≤ 0·050 *versus* healthy control; †*P* ≤ 0·050 (Kruskal–Wallis test).

Coagulopathy (as assessed by the international normalized ratio) and decreased lymphocyte and monocyte counts were related to increasing severity. Biomarker levels showed a generalized trend to increase with disease severity (*Figs* 1 and 2; *Table* *S3*, supporting information).

Confounding factors from *Table* 1 that yielded *P* ≤ 0·100 in univariable analysis, to be introduced as adjusting variables in multivariable analyses, are shown in *Table* *S4* (supporting information).

Multivariable analysis of biomarker levels to evaluate the risk of sepsis *versus* infection identified seven biomarkers of endothelial dysfunction, two of neutrophil degranulation and 13 of emergency granulopoiesis as independent risk factors for sepsis (*Table* 2).

**Table 2:** Multivariable analysis for risk of sepsis *versus* infection

Biomarker*	Indicator for	Odds ratio	*P*	Benjamini–Hochberg *P*
Mid‐regional proadrenomedullin (nmol/l)	ED	11·53 (4·15, 32·08)	< 0·001	0·006
Syndecan 1 (pg/ml)	ED	9·48 (2·86, 31·38)	< 0·001	0·006
Thrombomodulin (pg/ml)	ED	4·14 (1·28, 13·39)	0·018	0·040
Angiopoietin 2 (pg/ml)	ED	3·70 (1·80, 7·59)	< 0·001	0·006
Endothelial cell‐specific molecule 1 (pg/ml)	ED	3·58 (1·45, 8·83)	0·006	0·022
Vascular cell adhesion molecule 1 (pg/ml)	ED	2·72 (1·10, 6·76)	0·031	0·047
E‐selectin (pg/ml)	ED	2·32 (1·12, 4·81)	0·023	0·041
Lipocalin 2 (pg/ml)	ND	2·27 (1·16, 4·44)	0·016	0·040
MMP8 (pg/ml)	ND	1·90 (1·23, 2·96)	0·004	0·016
Procalcitonin (ng/ml)	AR	1·83 (1·41, 2·37)	< 0·001	0·006
Chitinase 1 (*CHIT1*) (copies/ng)	EG	1·81 (1·24, 2·64)	0·002	0·009
Stomatin (*STOM*) (copies/ng)	EG	1·68 (1·09, 2·60)	0·020	0·040
MMP9 (*MMP9*) (copies/ng)	EG	1·67 (1·14, 2·44)	0·008	0·026
Interleukin‐1 receptor type 2 (*IL1R2*) (copies/ng)	EG	1·64 (1·12, 2·42)	0·011	0·006
MMP8 (*MMP8*) (copies/ng)	EG	1·64 (1·23, 2·19)	0·001	0·033
Lipocalin 2 (*LCN2*) (copies/ng)	EG	1·62 (1·23, 2·15)	0·001	0·006
Transcobalamin 1 (*TCN1*) (copies/ng)	EG	1·56 (1·07, 2·27)	0·021	0·040
Lactoferrin (*LTF*) (copies/ng)	EG	1·55 (1·16, 2·06)	0·002	0·009
Bactericidal/permeability‐increasing protein (*BPI*) (copies/ng)	EG	1·52 (1·07, 2·17)	0·020	0·040
CD24 (*CD24*) (copies/ng)	EG	1·51 (1·05, 2·17)	0·026	0·043
C‐reactive protein (mg/l)	AR	1·51 (1·05, 2·18)	0·028	0·044
MMP25 (*MMP25*) (copies/ng)	EG	1·46 (1·05, 2·05)	0·026	0·043
CD177 (*CD177*) (copies/ng)	EG	1·31 (1·05, 1·65)	0·020	0·040
Olfactomedin 4 (*OLFM4*) (copies/ng)	EG	1·28 (1·05, 1·55)	0·012	0·033

Values in parentheses are 95 per cent confidence intervals.

* Biomarker values correspond to napierian logarithms. Variables adjusted for gene expression biomarkers were age, cardiovascular disease, immunosuppression, high BP, chronic respiratory disease, chronic renal disease, abdominal surgery, other surgery, respiratory source of infection and abdominal source of infection; variables adjusted for protein biomarkers were age, immunosuppression, high BP, chronic respiratory disease, chronic renal disease, urgent surgery, abdominal surgery, other surgery, respiratory source of infection and abdominal source of infection (*Table* *S4*, supporting information). ED, endothelial dysfunction; ND, neutrophil degranulation; MMP, matrix metalloproteinase; AR, acute‐phase response; EG, emergency granulopoiesis; CD, cluster of differentiation.

Multivariable analysis to evaluate the risk of septic shock *versus* sepsis revealed four biomarkers of endothelial dysfunction, six of neutrophil degranulation and 14 of emergency granulopoiesis as independent risk factors for septic shock (*Table* 3).

**Table 3:** Multivariable analysis for risk of septic shock *versus* sepsis

Biomarker*	Indicator for	Odds ratio	*P*	Benjamini–Hochberg *P*
Proteinase 3 (pg/ml)	ND	8·09 (1·34, 48·91)	0·023	0·028
Lipocalin 2 (pg/ml)	ND	6·62 (2·47, 17·77)	< 0·001	0·002
Syndecan 1 (pg/ml)	ED	6·10 (1·77, 21·06)	0·004	0·006
Mid‐regional proadrenomedullin (nmol/l)	ED	4·58 (1·99, 10·58)	< 0·001	0·002
Thrombomodulin (pg/ml)	ED	4·52 (1·42, 14·34)	0·011	0·014
Interleukin‐18 receptor type 1 (*IL18R1*) (copies/ng)	ND	4·22 (2·26, 7·85)	< 0·001	0·002
Stomatin (*STOM*) (copies/ng)	EG	3·74 (1·87, 7·45)	< 0·001	0·002
Interleukin‐1 receptor type 2 (*IL1R2*) (copies/ng)	EG	3·72 (2·10, 6·58)	< 0·001	0·002
Angiopoietin 2 (pg/ml)	ED	3·02 (1·29, 7·10)	0·011	0·014
MMP8 (pg/ml)	ND	2·97 (1·55, 5·67)	0·001	0·002
MMP9 (*MMP9*) (copies/ng)	EG	2·67 (1·55, 4·59)	< 0·001	0·002
Lipocalin 2 (*LCN2*) (copies/ng)	EG	2·45 (1·71, 3·50)	< 0·001	0·002
MMP8 (*MMP8*) (copies/ng)	EG	2·43 (1·74, 3·38)	< 0·001	0·002
Transcobalamin 1 (*TCN1*) (copies/ng)	EG	2·36 (1·62, 3·44)	< 0·001	0·002
Lactoferrin (pg/ml)	ND	2·30 (1·38, 3·84)	0·001	0·002
Myeloperoxidase (pg/ml)	ND	2·26 (1·20, 4·25)	0·011	0·014
Lactoferrin (*LTF*) (copies/ng)	EG	2·24 (1·59, 3·15)	< 0·001	0·002
Bactericidal/permeability‐increasing protein (*BPI*) (copies/ng)	EG	2·23 (1·49, 3·36)	< 0·001	0·002
CD24 (*CD24*) (copies/ng)	EG	2·15 (1·47, 3·16)	< 0·001	0·002
Chitinase 1 (*CHIT1*) (copies/ng)	EG	2·01 (1·42, 2·83)	< 0·001	0·002
CD177 (*CD177*) (copies/ng)	EG	1·97 (1·30, 2·99)	0·001	0·002
Olfactomedin 4 (*OLFM4*) (copies/ng)	EG	1·85 (1·42, 2·40)	< 0·001	0·002
Carcinoembryonic antigen‐related cell adhesion molecule 8 (*CEACAM8*) (copies/ng)	EG	1·78 (1·31, 2·41)	< 0·001	0·002
Procalcitonin (ng/ml)	AR	1·73 (1·31, 2·29)	< 0·001	0·002
Myeloperoxidase (*MPO*) (copies/ng)	EG	1·36 (1·02, 1·81)	0·038	0·044

Values in parentheses are 95 per cent confidence intervals.

* Biomarker values correspond to napierian logarithms. Variables adjusted for gene expression biomarkers were age, abdominal surgery, abdominal source of infection, bacteraemia, other sources of infection, presence of Gram‐negative organisms and presence of polymicrobial infection; variables adjusted for protein biomarkers were surgical‐site source of infection, bacteraemia, presence of Gram‐negative organisms, and presence of polymicrobial infection (*Table* *S4*, supporting information). ND, neutrophil degranulation; ED, endothelial dysfunction; EG, emergency granulopoiesis; MMP, matrix metalloproteinase; CD, cluster of differentiation; AR, acute‐phase response.

**Figure 3:**
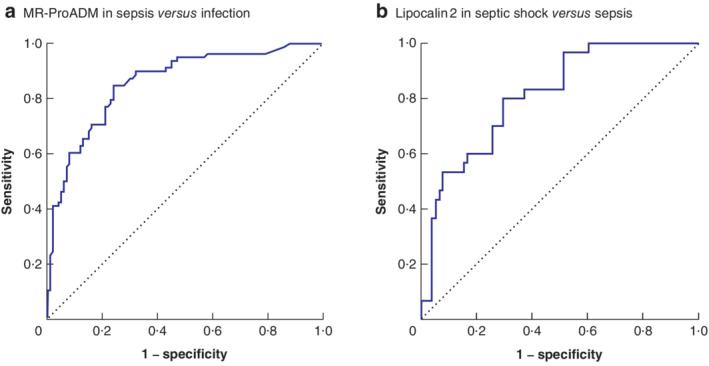
Area‐under‐the‐curve analysis evaluating the accuracy of two biomarkers in differentiating sepsis from infection or from septic shock **a** Accuracy of mid‐regional proadrenomedullin (MR‐ProADM) in differentiating sepsis from infection. Area under the curve (AUC): 0·86, 95 per cent c.i. 0·80 to 0·91 (*P* < 0·001); optimal operating point (OOP): 1·165 nmol/l; sensitivity: 84·6 per cent; specificity: 76·0 per cent. **b** Accuracy of lipocalin 2 in differentiating septic shock from sepsis. AUC: 0·81, 95 per cent c.i. 0·73 to 0·90 (*P* < 0·001); OOP: 246 346 pg/ml; sensitivity: 80·0 per cent; specificity: 70·5 per cent.

The AUC analysis to assess biomarker sensitivity and specificity indicated that mid‐regional proadrenomedullin (MR‐ProADM) was the best biomarker for differentiating sepsis from infection, whereas lipocalin 2 in plasma was the best biomarker for distinguishing septic shock from sepsis (*Fig*. 3).

## Discussion

This study found that a panel of seven biomarkers related to endothelial dysfunction (MR‐ProADM, syndecan (SDC) 1, thrombomodulin (THBD), angiopoietin (ANGPT) 2, endothelial cell‐specific molecule 1, vascular cell adhesion molecule 1 and E‐selectin) were associated with the presence of sepsis in patients with infection. This suggests that induction of endothelial injury is an early event as organ dysfunction develops.

SDC1 and MR‐proADM were the biomarkers showing the highest odds ratios for sepsis. SDC1 is a glycosaminoglycan shed from the endothelial glycocalyx during sepsis, and levels in plasma correlate with the SOFA score^20^, ^21^. In the present study, MR‐proADM was the biomarker of endothelial dysfunction showing not only the strongest association but also the best balance between sensitivity and specificity for sepsis, with an AUC of 0·86. Adrenomedullin is secreted from various organs and tissues, including vascular endothelial cells. It regulates vascular tone and endothelial permeability^22^. MR‐proADM, the mid‐regional fragment of proadrenomedullin, is more stable and directly reflects levels of the rapidly degraded active adrenomedullin peptide^23^. There is growing evidence of the value of MR‐ProADM as an early marker of severity in patients with infection^24^ and as a predictor of organ failure in patients with community‐acquired pneumonia^25^.

In the comparison of sepsis and septic shock, the number of biomarkers of endothelial dysfunction independently associated with septic shock dropped to four (SDC1, MR‐ProADM, THBD and ANGPT2). In contrast, six biomarkers denoting neutrophil degranulation were associated with septic shock: proteinase 3 (a serine protease), lipocalin 2 (a neutrophil gelatinase‐associated protein), interleukin‐18 receptor type 1 (an inductor of neutrophil degranulation)^26^, matrix metalloproteinase (MMP) 8 (a neutrophil collagenase), lactoferrin (a major iron‐binding protein) and myeloperoxidase (a heme protein). Only two of these biomarkers seemed relevant to differentiate sepsis from plain infection (lipocalin 2 and MMP8), suggesting that neutrophil degranulation may be important in the pathogenesis of septic shock. Proteins released from neutrophil granules could be mediating antibacterial effects^8^, ^27^, ^28^, ^29^, ^30^, and may participate in tissue remodelling^31^, attenuation of inflammation^32^ and preventing the deleterious effects of neutrophil extracellular traps^33^. However, increased intravascular levels of degranulated proteins could induce enhanced proteolysis^34^, endothelial injury and organ failure^35^, ^36^, ^37^, ^38^, ^39^.

Proteinase 3 and lipocalin 2 had strongest associations with the presence of septic shock. Neutrophil degranulation can lead to increased endothelial permeability via a mechanism that, in part, involves the actions of proteinase 3^40^, and a multimarker model containing proteinase 3 was able to predict the risk of septic acute kidney injury in patients with septic shock^41^. In the present study, lipocalin 2 was the marker showing the best balance between sensitivity and specificity in detecting septic shock. Lipocalin 2 has been used for risk stratification, early diagnosis and prognostication of sepsis in the emergency department^42^, ^43^. This protein is associated with mortality and multiple organ dysfunction syndrome in severe sepsis and septic shock^44^. Lipocalin 2 has been promoted as a relatively robust predictor of 28‐day mortality in severe sepsis^45^.

The present study has shown that emergency granulopoiesis is a preserved signature of both sepsis and septic shock, although to a greater degree in septic shock. The observed parallel between emergency granulopoiesis signatures and severity is in agreement with a previous study^9^ demonstrating that, in sepsis, the increased presence of circulating immature granulocytes is linked to clinical deterioration.

Regarding acute‐phase biomarkers, procalcitonin showed modest associations with the risk of sepsis and septic shock, while C‐reactive protein showed a mild association, exclusively with the risk of sepsis. These results indicate that neither procalcitonin nor C‐reactive protein is suitable for severity stratification in patients with infection.

Profiling protein levels in plasma of MR‐ProADM and lipocalin 2 could contribute to stratification of the severity of infection, particularly in settings where calculation of the SOFA score is not feasible. Evaluation of protein biomarkers is technically easier than evaluating those of transcriptomic nature. Emerging point‐of‐care devices could result in evaluation of these biomarkers in clinical practice as results can be obtained in less than 1 h^46^.

This study has an important limitation in that biomarkers were compared only at diagnosis of infection, sepsis or septic shock. Further prospective follow‐up studies with serial sampling should validate the potential role of MR‐ProADM and lipocalin 2 in predicting clinical worsening of patients with infection or sepsis.

## Supporting information


**Table S1.** Description of endothelial dysfunction biomarkers
**Table S2.** Description of emergency granulopoiesis biomarkers
**Appendix S1.** Methods for biomarker profiling
**Table S3.** Biomarker levels across groups
**Table S4.** Univariable analysis selecting confounding variables to be entered into multivariable analyses
