# Supplementary material for: Endothelial dysfunction is an early indicator of sepsis and neutrophil degranulation of septic shock in surgical patients
Source: BJS Open. 2020 Feb 19;4(3):524–34. doi: 10.1002/bjs5.50265 (PMC7260414; doi:10.1002/bjs5.50265)
Supplement: Supplementary file 1 — Table S1. Description of endothelial dysfunction biomarkers Table S2. Description of emergency granulopoiesis biomarkers Appendix S1. Methods for biomarker profiling Table S3. Biomarker levels across groups Table S4. Univariable analysis selecting confounding variables to be entered into multivariable analyses [file BJS5-4-524-s001.docx]

**BJS5_50265**

**Endothelial dysfunction is an early indicator of sepsis and neutrophil degranulation of septic shock in surgical patients**

**M. Martin-Fernandez, L. M. Vaquero-Roncero, R. Almansa, E. Gómez-Sánchez, S. Martín, E. Tamayo, M. C. Esteban-Velasco, P. Ruiz-Granado, M. Aragón, D. Calvo, J. Rico-Feijoo, A. Ortega, E. Gómez-Pesquera, M. Lorenzo-López, J. López, C. Doncel, C. González-Sanchez, D. Álvarez, E. Zarca, A. Ríos-Llorente, A. Diaz-Alvarez, E. Sanchez-Barrado, D. Andaluz-Ojeda, J. M. Calvo-Vecino, L. Muñoz-Bellvís, J. I. Gomez-Herreras, C. Abad-Molina, J. F. Bermejo-Martin, C. Aldecoa and M. Heredia-Rodríguez**

**Table S1** Description of endothelial dysfunction biomarkers

| **GENE** | **TYPE OF PROTEIN** | **PROTEIN FUNCTION** |
| --- | --- | --- |
| **Angiopoietin-1**  **(ANGPT1)** | Secreted glycoprotein | Inhibition of endothelial permeability |
| **Endothelial cell-specific molecule 1 (ESM1)** | Secreted protein | Promotes angiogenic sprouting |
| **Intercellular Adhesion Molecule 1**  **(ICAM-1)** | Cell surface glycoprotein | Its engagement promotes the assembly of endothelial apical cups during leukocyte trans-endothelial migration |
| **E-Selectin**  **(SEL-E)** | Cell surface glycoprotein | Mediates in the adhesion of blood neutrophils in cytokine-activated endothelium |
| **P-Selectin**  **(SEL-P)** | Ca(2+)-dependent receptor | Mediates rapid rolling of leukocyte rolling over vascular surfaces during the initial steps in inflammation |
| **Syndecan-1**  **(SDC1)** | Transmembrane proteoglycan | Cell proliferation, cell migration and cell-matrix interactions |
| **Vascular Cell Adhesion Molecule 1**  **(VCAM-1)** | Cell surface sialoglycoprotein | Leukocyte-endothelial cell adhesion and signal transduction |
| **Angiopoietin-2**  **(ANGPT2)** | Secreted glycoprotein | Disruption of the vascular remodeling ability of ANGPT1 and induction of endothelial cell apoptosis |
| **Thrombomodulin**  **(THBD)** | Endothelial-specific type I membrane receptor | Reduction of the amount of thrombin generated |
| **Mid-Regional** **Proadrenomedullin**  **(MR-ProADM)** | Secreted protein | Vasodilatory, anti-inflammatory and antimicrobial activity |

**Table S2** Description of emergency granulopoiesis biomarkers

| **GENE** | **CELL** | **PROTEIN LOCATION** |
| --- | --- | --- |
| STOM  BPI  MPO  ELANE  AZU1  PRTN3  CTSG  CEACAM6  DEFA4 | **Promyelocyte** | **Azurophil granules** |
| STOM  TCN1  LTF  CHIT1  MMP8  LCN2  MMP9  OLFM4  CD177 | **Myelocytes and metamyelocytes** | **Specific granules** |
| MMP9  CEACAM8  CD177 | **Band cells** | **Gelatinase granules in band cells** |
| STOM  MMP25 | **Segmented neutrophilic cells** | **Secretory vesicles** |
| STOM  CD24  IL1R2  IL18R1 | **Myelocytes and metamyelocytes** | **Plasmatic membrane** |

**Appendix S1** Methods for biomarker profiling

**Quantification of endothelial dysfunction biomarkers in plasma:** MR-proADM measurement was performed by TRACE technology (Time Resolved Amplified Cryptate Emission) using a sandwich immunoassay (Kryptor Compact Plus Analyser, BRAHMS, Hennigsdorf, Germany). Quantification of levels of Angiopoietin 1 (ANGPT1) and 2 (ANGPT2), Endocan (ESM1), Intercellular Adhesion Molecule 1 (ICAM-1), E-Selectin (SEL-E), P-Selectin (SEL-P), Syndecan 1 (SDC1), Vascular Cell Adhesion Molecule 1 (VCAM-1) and Thrombomodulin (THBD) was performed on a Luminex 200 platform by using reagents from R&D as per manufacturer`s protocol. Samples from 207 patients were available for these analyses.

**Quantification of neutrophil degranulation biomarkers in plasma:** levels of LCN2/NGAL, MMP8, LTF, PRTN3, MPO and MMP9 were quantified on a Luminex 200 platform by using reagents from R&D as per manufacturer`s protocol. Samples from 207 patients were available for these analyses.

**Quantification of transcriptomic biomarkers of emergency granulopoiesis:** genes encoding proteins stored in the neutrophil granules were evaluated for their expression levels in blood. Genes were selected from the results of our EXPRESS (Gene Expression in Sepsis) study (PMID: 25557485), which compared gene expression profiles of surgical patients with and without sepsis (microarray data from this study are available at the Array Express microarray data repository with code E-MTAB-1548). 2.5 mL of blood were collected by using PaxGene (BD) venous blood vacuum collection tubes. Total RNA was extracted from blood samples using the PAXgene Blood RNA System (PreAnalytix, Hombrechtikon, Switzerland). The evaluation of concentration and quality was performed by spectrometry (Nano-Drop ND1000, NanoDrop Technologies,Wilmington, DE). cDNA was generated from each sample on a Techne TC-512 thermal cycler (Bibby-Scientific, Staffordshire, OSA, UK) starting from 1000 ng of mRNA by using iScript Advanced cDNA Synthesis Kit (BioRad, cat:1725038). The obtained volume of cDNA (20 uL) was further diluted (1/25), and 2.5 uL (5 ng of total mRNA) were employed for quantification of target gene expression using ddPCR on a BioRad QX200 platform (California, USA), according to the manufacturer instructions. Expression of the genes selected for the study was quantified using predesigned TaqMan Assay Primer/Probe Sets, (FAM or VIC labeled MGB probes, Thermo Fisher/Scientific-Life Technologies, Waltham, MA): Matrix metallopeptidase 8 (MMP8): Hs01029057_m1; Lactotransferrin (LTF): Hs00914334_m1; Proteinase 3 (PRTN3):Hs01597752_m1; lipocalin 2/ neutrophil gelatinase associated lipocalin (LCN2/NGAL): Hs01008571_m1; Olfactomedin 4 (OLFM4): Hs00197437_m1; Elastase (ELANE): Hs00236952_m1; Myeloperoxidase (MPO): Hs00924296_m1; cathepsin G (CTSG): Hs00175195_m1; azurocidin 1 (AZU1): Hs00156049_m1; defensin alpha 4 (DEFA4): Hs00157252_m1; Bactericidal / permeability-increasing protein (BPI): Hs01552756_m1; Interleukin 18 Receptor 1 (IL18R1): Hs00977691_m1; Carcinoembryonic antigen related cell adhesion molecule 8 (CEACAM8): Hs00266198_m1; Carcinoembryonic antigen related cell adhesion molecule 6 (CEACAM6): Hs03645554_m1; Small Cell Lung Carcinoma Cluster 4 Antigen (CD24): Hs02379687_s1; transcobalamin 1 (TCN1): Hs01055542_m1; Stomatin (STOM): Hs00925242_m1; Interleukin 1 Receptor Type 2 (IL1R2): Hs00174759_m1; Cluster of Differentiation molecule 177 molecule (CD177): Hs00360669_m1; chitinase 1 (CHIT1): Hs00185753_m1; matrix metallopeptidase 9 (MMP9): Hs00957562_m1; matrix metallopeptidase 25 (MMP25): Hs00360861_m1. Briefly, ddPCR was performed using ddPCR Supermix for Probes (no dUTP), and BioRad standard reagents for droplet generation and reading. End-point PCR with 40 cycles was performed by using C1000Touch Thermal Cycler (BioRad) after splitting each sample into approximately 20,000 droplets. Next, the droplet reader used at least 10,000 droplets to determine the percentage of positive droplets and calculation of copy number of cDNA per nanogram of initial mRNA. mRNA from the 252 patients were available for this analysis.

**Quantification of Procalcitonin and C-reactive Protein in plasma**: procalcitonin (PCT) measurement was performed by electrochemiluminescence immunoassay on a chemistry analyzer (Cobas 6000, Roche Diagnostics, Meylan, France) limit of detection 0.02 ng/mL. Serum CRP and lactate were measured by particle enhanced immunoturbidimetric and colorimetric assay, respectively (e501 Module Analyser, Roche Diagnostics, Meylan, France); limit of detection 0.15 mg/dL and 0.2 mmol/L, respectively. Samples from the 252 patients were available for these analyses.

**Table S3** Biomarker levels across groups

|  | **Healthy Controls**  **(0)** | **Infection**  **(1)** | **Sepsis**  **(2)** | **Septic Shock**  **(3)** | **p**  **(0 vs 1)** | **p**  **(0 vs 2)** | **p**  **(0 vs 3)** | **P**  **(1 vs 2)** | **p**  **(1 vs 3)** | **p**  **(2 vs 3)** |
| --- | --- | --- | --- | --- | --- | --- | --- | --- | --- | --- |
| **MMP8 (c/ng)** | 18  [53] | 383  [1001] | 824  [2 689] | 10 280  [19 432] | 0.001 | < 0.001 | < 0.001 | n.s. | < 0.001 | < 0.001 |
| **LTF (c/ng)** | 38  [169] | 126  [209] | 476  [1 408] | 3 184  [4 730] | n.s. | 0.008 | < 0.001 | < 0.001 | < 0.001 | < 0.001 |
| **PRTN3 (c/ng)** | 3  [4] | 9  [25] | 28  [104] | 77  [294] | n.s. | 0.046 | < 0.001 | < 0.001 | < 0.001 | 0.026 |
| **LCN2 (c/ng)** | 173  [354] | 321  [806] | 1676  [4 814] | 13 400  [22 307] | n.s. | 0.001 | < 0.001 | < 0.001 | < 0.001 | < 0.001 |
| **OLFM4 (c/ng)** | 11  [15] | 29  [106] | 170  [825] | 1 600  [3 138] | 0.010 | 0.001 | < 0.001 | < 0.001 | < 0.001 | < 0.001 |
| **ELANE (c/ng)** | 10  [10] | 31  [49] | 56  [123] | 103  [308] | 0.043 | 0.008 | < 0.001 | < 0.001 | < 0.001 | n.s. |
| **MPO (c/ng)** | 14  [12] | 26  [31] | 37  [107] | 94  [186] | n.s. | 0.046 | 0.001 | 0.012 | < 0.001 | 0.001 |
| **CTSG (c/ng)** | 15  [17] | 17  [24] | 37  [102] | 55  [215] | n.s. | 0.008 | 0.005 | 0.001 | < 0.001 | n.s. |
| **AZU1 (c/ng)** | 9  [6] | 17  [19] | 31  [111] | 56  [151] | n.s. | 0.014 | 0.001 | < 0.001 | < 0.001 | n.s. |
| **DEFA4 (c/ng)** | 74  [112] | 70  [151] | 142  [537] | 310  [984] | n.s. | n.s. | < 0.001 | 0.012 | < 0.001 | n.s. |
| **BPI (c/ng)** | 48  [75] | 95  [222] | 292  [516] | 752  [872] | n.s. | 0.001 | 0.001 | < 0.001 | < 0.001 | < 0.001 |
| **IL18R1 (c/ng)** | 85  [21] | 177  [366] | 356  [712] | 1 036  [658] | 0.001 | < 0.001 | < 0.001 | 0.031 | < 0.001 | < 0.001 |
| **CEACAM8 (c/ng)** | 23  [69] | 38  [62] | 85  [289] | 314  [697] | n.s. | n.s. | 0.001 | 0.002 | < 0.001 | < 0.001 |
| **CEACAM6 (c/ng)** | 8  [13] | 12  [14] | 24  [88] | 70  [107] | n.s. | 0.046 | 0.001 | 0.008 | < 0.001 | 0.009 |
| **CD24 (c/ng)** | 114  [77] | 105  [123] | 201  [424] | 750  [782] | n.s. | 0.046 | 0.001 | 0.005 | < 0.001 | < 0.001 |
| **TCN1 (c/ng)** | 49  [32] | 62  [87] | 148  [332] | 588  [869] | n.s. | 0.001 | < 0.001 | < 0.001 | < 0.001 | < 0.001 |
| **STOM (c/ng)** | 372  [118] | 718  [1175] | 1 688  [2 236] | 3 416  [2 520] | 0.001 | < 0.001 | < 0.001 | < 0.001 | < 0.001 | < 0.001 |
| **IL1R2 (c/ng)** | 326  [195] | 940  [2 157] | 2 860  [4 820] | 8 628  [6 388] | < 0.001 | < 0.001 | < 0.001 | < 0.001 | < 0.001 | < 0.001 |
| **CD177 (c/ng)** | 15  [27] | 1 286  [5 228] | 4 460  [9 864] | 10 156  [10 122] | 0.001 | < 0.001 | < 0.001 | 0.002 | < 0.001 | 0.009 |
| **CHIT1 (c/ng)** | 8  [7] | 13  [16] | 36  [83] | 152  [250] | n.s. | 0.001 | < 0.001 | < 0.001 | < 0.001 | < 0.001 |
| **MMP9 (c/ng)** | 662  [681] | 2 508  [5 552] | 7 356  [9 808] | 14 960  [15 366] | < 0.001 | < 0.001 | < 0.001 | 0.001 | < 0.001 | 0.026 |
| **MMP25 (c/ng)** | 37  [25] | 132  [299] | 380  [681] | 464  [675] | 0.001 | < 0.001 | < 0.001 | 0.002 | < 0.001 | n.s. |
| **MR-ProADM (nmol/L)** | 0.46  [0.11] | 0.73  [0.66] | 2.32  [2.16] | 5.01  [4.44] | < 0.001 | < 0.001 | < 0.001 | < 0.001 | < 0.001 | 0.016 |
| **ANGPT1 (pg/mL)** | 33 755  [22 623] | 10 621  [11 941] | 8 898  [9 321] | 12 548  [14 264] | 0.005 | 0.005 | 0.021 | n.s. | n.s. | n.s. |
| **ESM1 (pg/mL)** | 858  [348] | 1 006  [522] | 1 241  [1 656] | 1 868  [1 904] | n.s. | n.s. | 0.002 | n.s. | 0.005 | n.s. |
| **ICAM-1 (pg/mL)** | 327 374  [127 446] | 428 988  [368 494] | 540 875  [392 835] | 635 785  [330 915] | n.s. | 0.043 | 0.021 | n.s. | 0.005 | n.s. |
| **SEL-E (pg/mL)** | 33 762  [13 322] | 41 472  [34 520] | 50 577  [58 245] | 49 529  [49 539] | n.s. | n.s. | n.s. | n.s. | n.s. | n.s. |
| **SDC1 (pg/mL)** | 3 014  [829] | 3 513  [1 812] | 6 120  [3 638] | 6 743  [5 877] | n.s. | 0.005 | 0.002 | < 0.001 | < 0.001 | n.s. |
| **VCAM-1 (pg/mL)** | 898 138  [197 691] | 1 491 250  [1 578 423] | 2 638 300  [2 232 050] | 3 412 550  [ 1430 300] | 0.048 | 0.005 | 0.002 | < 0.001 | < 0.001 | n.s. |
| **ANGPT2 (pg/mL)** | 3 636  [6 719] | 3 296  [4 140] | 8 418  [6 689] | 12 063  [14 661] | n.s. | n.s. | n.s. | < 0.001 | < 0.001 | n.s. |
| **SEL-P (pg/mL)** | 64 218  [17 822] | 26 376  [10 681] | 32 493  [12 373] | 38 663  [25 477] | 0.005 | 0.005 | 0.002 | 0.039 | n.s. | n.s. |
| **THBD (pg/mL)** | 7 947  [3 254] | 6 584  [3 627] | 9 144  [6 290] | 12 214  [11 893] | n.s. | 0.043 | 0.002 | < 0.001 | 0.001 | n.s. |
| **MMP8 (pg/mL)** | 3 494  [6 115] | 3 519  [8 726] | 10 589  [16 273] | 29 003  [30 652] | n.s. | n.s. | 0.002 | < 0.001 | < 0.001 | < 0.001 |
| **LTF (pg/mL)** | 143 137  [197 587] | 69 638  [133 645] | 137 668  [210 631] | 349 465  [578 538] | n.s. | n.s. | n.s. | 0.039 | < 0.001 | 0.004 |
| **PRTN3 (pg/mL)** | 27 001  [26 638] | 40 433  [32 985] | 57 355  [32 694] | 76 000  [11 819] | n.s. | 0.043 | 0.002 | 0.002 | < 0.001 | 0.004 |
| **LCN2 (pg/mL)** | 75 706  [24 713] | 83 571  [58 129] | 200 834  [161 095] | 437 898  [410 121] | n.s. | 0.005 | 0.002 | < 0.001 | < 0.001 | < 0.001 |
| **MMP9 (pg/mL)** | 90 675  [77 230] | 162 389  [185 533] | 151 520  [178 528] | 95 723  [152 227] | n.s. | n.s. | n.s. | n.s. | n.s. | n.s. |
| **MPO (pg/mL)** | 136 305  [197 837] | 64 873  [68 125] | 81 790  [70 588] | 127 514  [313 150] | n.s. | n.s. | n.s. | n.s. | n.s. | n.s. |
| **PCT (ng/mL)** | 0.03  [0.02] | 0.16  [0.57] | 2.00  [6.32] | 12.09  [34.84] | < 0.001 | < 0.001 | < 0.001 | < 0.001 | < 0.001 | < 0.001 |
| **CRP (mg/L)** | 1.82  [3.04] | 117.00  [176.26] | 217.20  [194.97] | 273.53  [160.05] | < 0.001 | < 0.001 | < 0.001 | < 0.001 | < 0.001 | n.s. |

Data are expressed as median [IQR]. c/ng: copies of cDNA per ng of mRNA analyzed.

**Table S4** Univariable analysis selecting confounding variables to be entered into multivariable analyses

1. Sepsis *versus* Infection (mRNA)

|  | **Univariate analysis** | | |
| --- | --- | --- | --- |
|  | **OR** | **[CI 95%]** | ***p*** |
| Age | 1.04 | 1.02-1.06 | < 0.001 |
| Cardiovascular disease | 2.25 | 1.08-4.71 | 0.031 |
| Immunosuppression | 2.99 | 1.02-8.73 | 0.046 |
| High blood pressure | 2.19 | 1.21-3.94 | 0.009 |
| Chronic respiratory disease | 7.53 | 1.65-34.35 | 0.009 |
| Chronic renal disease | 4.97 | 1.05-23.65 | 0.044 |
| Abdominal surgery | 0.30 | 0.15-0.61 | 0.001 |
| Other surgeries | 0.23 | 0.07-0.73 | 0.012 |
| Respiratory source of infection | 4.38 | 1.39-13.73 | 0.011 |
| Abdominal source of infection | 0.45 | 0.25-0.81 | 0.008 |

1. Sepsis *versus* Infection (prot)

|  | **Univariate analysis** | | |
| --- | --- | --- | --- |
|  | **OR** | **[CI 95%]** | ***p*** |
| Age | 1.04 | 1.02-1.06 | < 0.001 |
| Immunosuppression | 3.40 | 1.14-10.11 | 0.028 |
| High blood pressure | 2.20 | 1.19-4.09 | 0.013 |
| Chronic respiratory disease | 6.91 | 1.47-32.56 | 0.014 |
| Chronic renal disease | 4.63 | 0.93-22.98 | 0.061 |
| Urgent surgery | 0.48 | 0.20-1.11 | 0.085 |
| Abdominal surgery | 0.30 | 0.15-0.62 | 0.001 |
| Other surgeries | 0.27 | 0.09-0.88 | 0.030 |
| Respiratory source of infection | 3.81 | 1.16-12.49 | 0.027 |
| Abdominal source of infection | 0.43 | 0.23-0.80 | 0.008 |

1. Septic shock *versus* Sepsis (mRNA)

|  | **Univariate analysis** | | |
| --- | --- | --- | --- |
|  | **OR** | **[CI 95%]** | ***p*** |
| Age | 1.02 | 1.00-1.05 | 0.091 |
| Abdominal surgery | 0.55 | 0.28-1.07 | 0.080 |
| Abdominal source of infection | 0.50 | 0.25-0.99 | 0.045 |
| Bacteremia | 4.05 | 1.42-11.52 | 0.009 |
| Other sources of infection | 0.38 | 0.12-1.19 | 0.097 |
| Presence of Gram - | 3.14 | 1.58-6.25 | 0.001 |
| Presence of polymicrobial infection | 3.17 | 1.51-6.62 | 0.002 |

1. Septic shock *versus* Sepsis (prot)

|  | **Univariate analysis** | | |
| --- | --- | --- | --- |
|  | **OR** | **[CI 95%]** | ***p*** |
| Surgical source of infection | 3.45 | 1.30-9.17 | 0.013 |
| Bacteremia | 4.73 | 1.36-16.47 | 0.015 |
| Presence of Gram - | 2.19 | 0.91-5.27 | 0.079 |
| Presence of polymicrobial infection | 2.75 | 1.07-7.09 | 0.036 |
